# Effects of Inpatient Multicomponent Occupational Rehabilitation versus Less Comprehensive Outpatient Rehabilitation on Somatic and Mental Health: Secondary Outcomes of a Randomized Clinical Trial

**DOI:** 10.1007/s10926-016-9679-5

**Published:** 2016-11-04

**Authors:** Lene Aasdahl, Kristine Pape, Ottar Vasseljen, Roar Johnsen, Sigmund Gismervik, Chris Jensen, Marius Steiro Fimland

**Affiliations:** 1 Department of Public Health and General Practice, Faculty of Medicine, NTNU, Norwegian University of Science and Technology, Trondheim, Norway; 2 Department of Physical Medicine and Rehabilitation, St. Olavs Hospital, Trondheim University Hospital, Trondheim, Norway; 3 National Center for Occupational Rehabilitation, Rauland, Norway; 4 Hysnes Rehabilitation Center, St. Olavs Hospital, Trondheim University Hospital, Trondheim, Norway

**Keywords:** Return to work, Sick leave, Musculoskeletal diseases, Absenteeism, Cognitive therapy

## Abstract

*Purpose* To evaluate effects on somatic and mental health of a multicomponent inpatient occupational rehabilitation program compared to a less comprehensive outpatient program in individuals on sick leave for musculoskeletal complaints or mental health disorders. *Methods* A randomized clinical trial with parallel groups. Participants were individuals on sick-leave for 2–12 months with a sick-leave diagnosis within the musculoskeletal, psychological or general and unspecified chapters of ICPC-2. Potential participants were identified in the Social Security System Registry. The multicomponent inpatient program (4 + 4 days) consisted of Acceptance and Commitment Therapy, physical training and work-related problem-solving including creating a return to work plan and a workplace visit if considered relevant. The comparative outpatient program consisted primarily of ACT (6 sessions during 6 weeks). Self-reported health-related quality of life, subjective health complaints, pain and anxiety and depression symptoms were assessed up to 12 months after the program. *Results* 168 individuals were randomized to the multicomponent inpatient program (n = 92) or the outpatient program (n = 76). Linear mixed models showed no statistically significant differences between the programs, except for slightly more reduced pain after the outpatient program. *Conclusions* This study presents no support that a 4 + 4 days multicomponent inpatient rehabilitation program is superior to a less comprehensive outpatient program, in improving health outcomes.

**Electronic supplementary material:**

The online version of this article (doi:10.1007/s10926-016-9679-5) contains supplementary material, which is available to authorized users.

## Introduction

Musculoskeletal and mental health disorders are the two leading causes of sickness absence in Norway [1]. Five percent of the gross domestic product is spent on disability and sickness benefits, and this is by far the highest level in the OECD countries [2].

Most occupational rehabilitation programs described in the scientific literature are directed towards specific diagnostic groups, mainly musculoskeletal disorders [3, 4]. Effects reported in the literature are ambiguous. For example, Jensen et al. [5] did not find added effects on return to work or pain reduction of multidisciplinary occupational rehabilitation compared to a brief intervention program for subjects with low back pain. In contrast, Lambeek et al. [6] and Loisel et al. [7] reported that multidisciplinary occupational rehabilitation led to increased return to work and reduced disability, but with little effect on pain. Others have found increased return to work rates, but no effect on functional status [8]. Studies on the effect of return to work programs for individuals with mental health disorders have also showed increased return to work, but no added reduction in symptoms [9, 10]. However, a recent study showed that work-focused cognitive behavioral therapy for individuals on sick leave with common mental disorders was more effective than usual care in reducing depression and anxiety symptoms, increasing health-related quality of life, as well as increasing or maintaining work participation [11].

In Norway, the occupational rehabilitation services offer both inpatient and outpatient programs to increase work participation and improve health outcomes for patients on sick-leave, and the inclusion of different diagnostic groups in the same rehabilitation programs has been common practice for several years [12]. However, effects of such programs have never been evaluated with a rigorous study design. Recently, we participated in developing a multicomponent occupational rehabilitation program [13]. The program consisted of cognitive behavioral therapy, physical training, creating a return to work plan, and a workplace visit if considered relevant by the participant and rehabilitation team. Physical exercise has been shown to reduce depression [14], seems to reduce pain [15] and is recommended as an adjunctive treatment for anxiety disorders [16]. Different diagnostic groups were included in the program. All activity at the center was framed within a cognitive behavioral therapy approach in the form of Acceptance and Commitment Therapy (ACT) [17]. The ACT model emphasizes accepting both negative and positive experiences, while focusing on a person`s values to guide them towards their goals [17]. In specific diagnostic groups there has been an increasing number of studies on the effect of ACT [18]. Although there are some inconsistencies in the literature [18, 19], studies suggest that ACT may have beneficial effects on chronic pain [20], anxiety [21, 22] and depression [21, 23].

We evaluated the effects of the multicomponent program delivered at the inpatient occupational rehabilitation center by comparing it to a less comprehensive outpatient program consisting mainly of ACT. In a recent study with 12 months of follow-up we found no difference between the programs on number of sickness absence days and return to work (under review). Here, we present results of secondary outcomes related to health as the programs also aimed to improve the participants` health status and health perception.

We hypothesized that the inpatient program, to a greater extent than the outpatient program, would reduce pain, depression, anxiety and subjective health complaints and increase function and health-related quality of life.

## Methods

### Study Design and Participants

We conducted a randomized clinical trial with parallel groups, comparing an inpatient multicomponent program (4 + 4 days) with a single-component program (6 sessions during 6 weeks) (hereafter referred to as the inpatient- and outpatient program, respectively) for individuals on sick-leave due to musculoskeletal-, unspecific-, or common mental health disorders. Details about the study design have been published elsewhere [13]. The primary outcome in the main study was sickness absence (under review). The current study assesses effects on somatic and mental health in the inpatient program versus the outpatient program through 12 months follow-up. The study was approved by the Regional Committee for Medical and Health Research Ethics in Central Norway (No.: 2012/1241), and the trial is registered in clinicaltrials.gov (No.: NCT01926574). The results are presented according to the CONSORT statement [24].

Eligible participants were individuals aged 18 to 60 years sick listed 2–12 months with a diagnosis within the musculoskeletal (L), psychological (P) or general and unspecified (A) chapters of ICPC-2 (International Classification of Primary Care, Second edition). Sick leave status at inclusion had to be at least 50 % off work. Exclusion criteria, assessed by a questionnaire and an outpatient screening performed by a physician, a physiotherapist and a psychologist, were: (1) alcohol or drug abuse; (2) serious somatic (e.g. cancer, unstable heart disease) or psychiatric disorders (e.g. high suicidal risk, psychosis, ongoing manic episode); (3) specific disorders requiring specialized treatment; (4) pregnancy; (5) currently participating in another treatment or rehabilitation program; (6) insufficient oral or written Norwegian language skills to participate in group sessions and fill out questionnaires; (7) scheduled for surgery within the next 6 months; and (8) serious problems with functioning in a group setting.

Data was obtained by questionnaires and filled out at six time-points: at screening before inclusion, at the start of the program, at the end of the program, and three, six and 12 months after the inpatient program ended.

### Programs

#### The Inpatient Program

Consisted of group discussions (ACT based) led by team coordinators, individual and group based physical training, mindfulness, psychoeducation on stress and individual meetings with coordinator for work-related problem- solving and creating a return to work plan. The intervention lasted four full workdays in week 1 and week 4 (8 days in total; 6–7 h each day), separated by 2 weeks at home (week 2 and 3). The two weeks at home included at least two contacts with the team coordinator (in person or by telephone) and a meeting with the employer if regarded relevant and the participant gave permission. A certified ACT instructor supervised the coordinators who mentored the participants before and during (monthly) the intervention. The program took place at Hysnes rehabilitation center, established as a part of St. Olavs Hospital, in central Norway. A more detailed description of the program has been published elsewhere [13].

#### The Outpatient Program

Consisted of group based ACT. The sessions were held at the Department of Physical Medicine and Rehabilitation at St. Olavs Hospital once a week for six weeks, each session lasting 2.5 h. One of two physicians (specialists in Physical Medicine and Rehabilitation) or a psychologist, all three educated in ACT, led the sessions. The participants were encouraged to practise at home between sessions, including a daily 15 min audio-guided mindfulness practice. In addition the participants were offered two individual sessions with a social worker experienced in occupational rehabilitation and trained in ACT to clarify personal values and work-related issues. The intervention also included a motivational group discussion with a physiotherapist on the benefits of physical training. An individual session with both the social worker and ACT therapist present ended the intervention. In this session a summary letter was written to the participant’s general practitioner. A more detailed description of the program has been published elsewhere [13].

### Outcome Measures

Self-reported data on health and functioning were collected via internet-based questionnaires. The participants received text messages on their mobile telephone when it was time to answer questionnaires and as reminders if they did not respond. If they had not responded after two text-message reminders a project co-worker made a final phone call to remind the participant.

Anxiety and depression were recorded using The Hospital Anxiety and Depression Scale (HADS) [25]. It consists of 14 items, where seven items measure anxiety and seven measure depression symptoms. It is scored on a 4-point Likert scale according to intensity of symptoms the last week. The maximum score is 21 on each subscale. HADS is widely used and has been found to perform well in assessing severity and detecting anxiety and depression, with a cut-off of 8 giving an optimal balance between sensitivity and specificity [26]. HADS was answered at all time-points, except at six months.

Common somatic and mental health problems were recorded using The Subjective Health Complaints Inventory (SHC) [27], which registers complaints in five subscales: musculoskeletal pain, pseudoneurology, gastrointestinal problems, allergy and flu. It consists of 29 questions regarding complaints experienced the last month—each scored on a 4-point Likert scale from 0 “not at all” to 3 “serious”. A severity score can be reported for each subscale or as a total score (score range 0–87) [27]. The questionnaire was answered at the start of the program, and three and 12 months after the program.

To assess pain we used two questions from the Brief Pain Inventory (BPI) [28]. The participants were asked to grade the strongest and average pain during the last week on a 0 (no pain) to 10 (worst imaginable pain) numeric rating scale. The pain questions were answered at all time-points, except at 6 months.

Health-related quality of life was recorded using 15D [29]. It contains 15 dimensions covering physical, mental and social well-being and generates a total score ranging from 1 (no problem on any dimension) to 0 (being dead). It has been suggested that the generic minimal important change is ±0.015 and a large change is ±0.035 [30]. It should be noted that in the Alanne et al. study the cut-off for “slightly better” for pain and depression alone were 0.036 and 0.051, respectively. 15 D was answered at all time-points, except at screening and the end of the program.

Functioning was recorded using COOP/WONKA [31]. It offers a self-reporting assessment of function in six domains. We used four of the domains: physical fitness, feelings, daily activity and social activity. Each domain is scored on a 5-point Likert scale from 1 (no problems/not affected) to 5 (huge problems/considerably affected). Answers were used as a continuous score (range 1–5). It was included at all time-points, except at screening and six months.

Participants were asked to evaluate their general health on a 4-point Likert scale from 1 “poor” to 4 “very good”. The variable was analysed both dichotomized (poor/not very good vs. good/very good) and as a continuous score (range 1–4). The question was answered at all time-points, except at screening.

### Randomization and Blinding

**Fig. 1:**
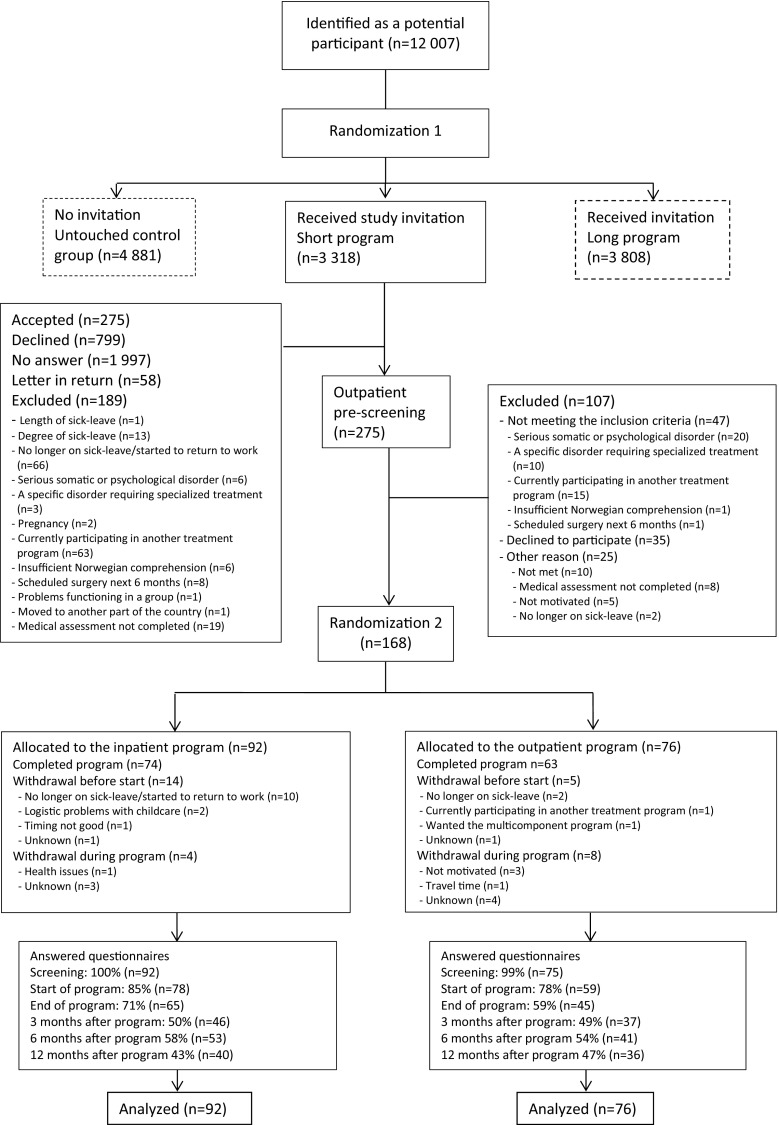
Participant flow through the study

The present study was part of a larger trial comparing the current (4 + 4 days) and a longer (3.5 weeks) inpatient program, an outpatient program, as well as a treatment as usual control group only followed in sick leave registers (see Fig. 1). The current study reports on health outcomes in the 4 + 4 days inpatient program and the comparative outpatient program.

Participants were randomized twice. Firstly, sick listed individuals identified in the Social Security System were randomized to receive an invitation to either the long or short program. Invited participants randomized to the short program completed a short initial questionnaire assessing eligibility. Those eligible were invited for an outpatient screening assessment. If the screening was passed (Fig. 1), the second randomization allocated the subjects to either the inpatient or the outpatient program. The first randomization was performed by a project co-worker. In the second allocation a flexibly weighted randomization procedure was provided by the Unit of Applied Clinical Research (third-party) at the Norwegian University of Science and Technology (NTNU), to ensure that the rehabilitation center had enough participants to run monthly groups in periods of low recruitment.

It was not possible to blind neither the participants nor the caregivers for treatment. Outcomes were measured using web-based questionnaires that the participants filled out independently on their own. The researchers were not blinded.

### Statistics

Sample size was calculated based on the primary outcome, i.e. number of sickness absence days (under review), resulting in 80 persons in each arm. Details about the estimations are published elsewhere [13].

Under the intention to treat principle we used linear (and logistic) mixed-effects models to compare outcome measures of health and function over time for the two rehabilitation programs. In addition to program and time (time points 1–6) we included an interaction term between program and the six time-points in the analyses to assess whether the effects of the programs differed over time. Repeated measurements (at the different time-points) were handled by including a random intercept for person in the models (thereby allowing the participants to start out at different levels) and a random slope for time (allowing individual development over time). The estimates from the analyses (fixed effects) were used to predict health outcomes at different time points for the two programs. We considered *p* values (two-tailed) <0.05 to be statistically significant. Precision was assessed using 95 % confidence intervals.

In the main analyses we did not adjust for baseline characteristics, but this was done in sensitivity analyses (gender, age, sick leave diagnosis, work status, education level and type of benefit) to assess the robustness of the results. Supplementary “per protocol” analyses were done by excluding participants that withdrew after randomization (before or during the programs) and/or attended less than 60 % of the sessions of the outpatient program.

Baseline characteristics for responders and non-responders to the 12 month follow-up questionnaire were compared using χ^2^ test, *t* test or Mann–Whitney U test. Median numbers of sickness absence days were compared by Mann–Whitney U test.

All analyses were done using STATA 13.1 (StataCorp. 2013. Stata Statistical Software: Release 13. College Station, TX: StataCorp LP).

## Results

In brief, 12 007 potential participants from the regional area were identified in the National Social Security System Registry. Of these, 3 318 were randomized to receive an invitation to the short program and 275 accepted. After screening 107 persons were excluded, withdrew or did not meet for their appointment. The remaining 168 persons were randomized to the inpatient program (n = 92) or the outpatient program (n = 76). The groups consisted of maximum 9 participants. The flow of participants through the study is illustrated in Fig. 1.

For the inpatient program, 14 people withdrew before they began the program and four quit during the program. For the outpatient program, five people withdrew before the program started and eight during the program. Those who started the outpatient program attended on average 7.9 of the 10 meetings and 59 (83 %) attended at least 60 % of the sessions. For the inpatient program there is no data for number of sessions participants attended, but as it was an inpatient program the participants were assumed compliant if they did not withdraw. All randomized participants were included in the analyses.

The number of people who answered the questionnaires decreased steadily through the study. For the inpatient program 100 % of the participants answered the questionnaire before the screening, 85 % at the start of program, 71 % at the end of the program, 50 % at three months, 58 % at six months and 43 % at 12 months after the program. For the outpatient program the numbers were 99, 78, 59, 49, 54 and 47 %, respectively. One participant in the outpatient program answered none of the questionnaires. At least 3 questionnaires were filled out by 72 % of the participants. A workplace visit was performed for 13 % of the participants randomized to the inpatient program.

### Participant Characteristics

The participants were mainly women (79 %), and their mean age was 45 years (SD 9.1) (Table 1). The majority (65 %) of the participants worked full-time prior to their sick leave, 18 % worked part time, 4 % had a graded disability pension and 13 % had no job. About half were on full sick-leave (45 %) and half on graded sick-leave (48 %). A smaller part (7 %) received work assessment allowance, which can be applied for in Norway after being on sick leave for a year. The latter group consisted of individuals who were invited to the study just before their benefit was changed from sick-leave to work assessment allowance. The median number of days on sick-leave the last 12 months before inclusion in the study (i.e. second randomization) was 226 days (interquartile range (IQR) 189–271). Sick-leave diagnoses within the musculoskeletal chapter in ICPC-2 were most common (52 %), followed by psychological (38 %) and general and unspecified (10 %). The baseline characteristics of the participants in the two programs were fairly similar (Table 1).

**Table 1:** Baseline characteristics of participants^a^

	Inpatient program (n = 92)	Outpatient program (n = 76)
Age mean (SD)	45.0 (8.7)	45.1 (9.6)
Women n (%)	71 (77 %)	62 (82 %)
Higher education^b^ n (%)	45 (49 %)	31 (41 %)
Work status n (%)		
No work	15 (16 %)	7 (9 %)
Full time	57 (62 %)	52 (68 %)
Part time	15 (16 %)	16 (21 %)
Graded disability pension	5 (5 %)	1 (1 %)
Sick-leave status^c^ n (%)		
Full sick-leave	41 (45 %)	35 (46 %)
Partial sick-leave	45 (49 %)	36 (47 %)
Work assessment allowance	6 (7 %)	5 (7 %)
Main diagnoses for sick-leave (ICPC-2)^c^ n (%)		
A-general and unspecified	9 (10 %)	7 (9 %)
L-musculoskeletal	48 (52 %)	40 (53 %)
P-psychological	35 (38 %)	29 (38 %)
Length of sick leave at inclusion^c,d^		
Median days (IQR)	224 (189–262)	229 (187–275)
HADS mean (SD)		
Anxiety (0–21)	7.8 (4.4)	7.4 (4.3)
Depression (0–21)	6.7 (4.3)	6.0 (4.1)
Pain level mean (SD)		
Average pain (0–10)	4.7 (2.3)	4.6 (2.0)
Strongest pain (0–10)	5.4 (2.5)	5.9 (2.0)
Quality of life 15D (0–1)		
Mean (SD)	0.79 (0.10)	0.79 (0.09)
Subjective health evaluation n (%)		
Poor	7 (8 %)	10 (13 %)
Not so good	55 (60 %)	39 (51 %)
Good	15 (16 %)	10 (13 %)
Very good	1 (1 %)	0
No response	14 (15 %)	17 (22 %)

^a^Work status, sick-leave status, diagnosis and length of sick leave recorded at inclusion. Education, HADS and pain recorded at screening. Quality of life and subjective health evaluation recorded at start of program

^b^Higher (tertiary) education (College or university)

^c^Based on data in the medical certificate from the National Social Security System Registry

^d^Number of days on sick leave during the last 12 months prior to inclusion. Measured as calendar days, not adjusted for graded sick- leave or part time job

### Outcome Measures

#### Comparison of Intervention Groups

**Fig. 2:**
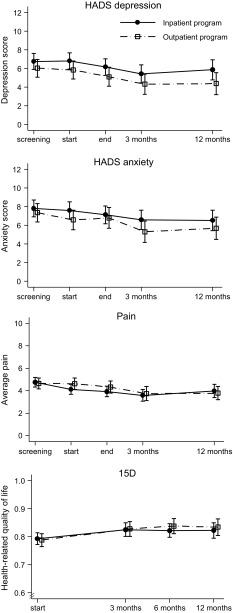
The Hospital Anxiety and Depression Scale (HADS), average pain and 15D. Data are estimated means with 95 % confidence intervals

Only one of the health measures, strongest pain, showed a statistically significant difference between the programs (Fig. 2 and Table 2). The estimated mean difference in strongest pain from start of the program to 12 months was 1.1 (95 % CI 0.1–2.0, *p* = 0.03) in favor of the outpatient program.

**Table 2:** Comparison of estimated health scores between the inpatient and the outpatient program

	Inpatient program		Outpatient program		Estimated difference between programs^b^		
	Mean	95 % CI	Mean	95 % CI	Mean	95 % CI	*p* value
HADS anxiety (0–21)							
Screening	7.8	6.9–8.7	7.3	6.3–8.3			
Start of program	7.6	6.7–8.5	6.6	5.5–7.6			
End of program	7.1	6.2–8.1	6.8	5.7–7.9			
3 months	6.6	5.5–7.6	5.3	4.2–6.5			
12 months	6.5	5.4–7.6	5.7	4.5–6.9	0.2	−1.2 to 1.5	0.78
HADS depression (0–21)							
Screening	6.7	5.9–7.6	6.0	5.1–7.0			
Start of program	6.8	5.9–7.7	5.8	4.9–6.8			
End of program	6.2	5.3–7.0	5.1	4.1–6.1			
3 months	5.4	4.5–6.4	4.3	3.2–5.4			
12 months	5.9	4.8–7.0	4.4	3.2–5.6	−0.5	−2.0 to 1.0	0.49
Average pain (0–10)							
Screening	4.8	4.3–5.2	4.6	4.2–5.1			
Start of program	4.1	3.7–4.6	4.6	4.1–5.1			
End of program	3.9	3.5–4.4	4.3	3.8–4.9			
3 months	3.6	3.1–4.1	3.8	3.2–4.4			
12 months	4.0	3.4–4.6	3.7	3.1–4.3	−0.8	−1.5 to 0.0	0.06
Strongest pain (0–10)							
Screening	5.4	4.9–5.9	6.0	5.5–6.5			
Start of program	5.0	4.5–5.5	6.0	5.5–6.6			
End of program	4.9	4.4–5.4	5.5	4.8–6.1			
3 months	4.7	4.1–5.3	4.9	4.2–5.5			
12 months	5.1	4.4–5.8	5.1	4.3–5.8	−1.1	−2.0 to −0.1	0.03
Health-related quality of life (0–1)							
Start of program	0.79	0.77–0.81	0.79	0.76–0.81			
3 months	0.82	0.80–0.85	0.83	0.80–0.85			
6 months	0.82	0.80–0.85	0.84	0.81–0.86			
12 months	0.82	0.80–0.85	0.83	0.80–0.86	−0.02	−0.05 to 0.02	0.41
SHC total^a^ (0–87)							
Start of program	15.2	13.4–16.9	15.9	13.9–17.9			
3 months	13.3	11.3–15.3	13.8	11.6–16.1			
12 months	14.3	12.2–16.5	13.9	11.6–16.2	−1.2	−3.8 to 1.5	0.39
SHC musculoskeletal pain^a^ (0–24)							
Start of program	6.0	5.2–6.8	6.7	5.7–7.6			
3 months	5.2	4.3–6.2	6.0	4.9–7.1			
12 months	5.7	4.7–6.7	5.5	4.4–6.6	−0.9	−2.1 to 0.4	0.17
SHC pseudoneurology (0–21)							
Start of program	5.0	4.3–5.7	5.1	4.3–5.9			
3 months	4.4	3.6–5.2	4.0	3.2–4.9			
12 months	4.4	3.6–5.2	4.8	4.0–5.6	0.3	−0.8 to 1.4	0.58
SHC gastrointestinal problems (0–21)							
Start of program	2.1	1.6–2.6	2.2	1.7–2.8			
3 months	1.8	1.2–2.3	2.1	1.4–2.7			
12 months	2.3	1.7–3.0	2.3	1.6–3.0	−0.3	−1.1 to 0.6	0.56

Means and mean differences with 95 % confidence intervals (95 % CI) were estimated using linear mixed models (unadjusted model)

^a^Estimates presented are from models without random slope due to lack of convergence

^b^Estimated from start of program to 12 months after the program; inpatient minus outpatient program

#### Development of Health Outcomes Over Time

Both programs showed increased health-related quality of life from start of the programs to 12 months (Table 2). The other health measures showed no or marginal changes (Table 2 and online supplementary Table 1).

#### Per Protocol, Sensitivity and Post Hoc Subgroup Analyses

The “per protocol” analyses provided only minor changes in the estimates. The estimated difference between the programs was statistically significant for average and strongest pain, in favour of the outpatient program. The main analyses were repeated adjusting for gender, age, diagnosis, education level, work status and type of benefit received. There were only small changes in the estimates and the adjusted analyses did not change any conclusions about the programs.

We performed subgroup analyses for HADS and average pain according to the two main diagnostic groups (see online supplementary Tables 2 and 3). For the HADS depression subscale there was a somewhat larger reduction in symptoms for participants with a psychological diagnosis. The same was observed for the HADS anxiety subscale for the inpatient program, while for the outpatient program there were only minor differences between the diagnostic groups. For average pain there was little difference between participants with a musculoskeletal- and psychological diagnosis for both programs. When performing the analyses for participants having the highest baseline scores on anxiety, depression and pain the results were similar to the main analyses. The differences between the two programs from start of the programs to 12 months were not statistically significant in any of the subgroup analyses.

#### Non-Responders

The participants not answering the questionnaire at 12 months were younger than the responders (mean age 43.6 (SD 9.3) vs. 46.7 (SD 8.6), *p* = 0.023). The other baseline values were fairly similar. The median number of sickness absence days during 12 months of follow-up were 87 (IQR 39–146) for the responders and 112 (IQR 44–185) for the non-responders (*p* = 0.252).

## Discussion

This randomized clinical trial showed no differences in self-reported health measures between a 4 + 4 days inpatient multicomponent occupational rehabilitation program and a less comprehensive outpatient program consisting mainly of group-based ACT, except for slightly more reduced pain after the outpatient program.

We are not aware of studies comparing inpatient and outpatient return to work programs. No substantial difference on somatic and mental health outcomes between the two rehabilitation programs is in line with some earlier studies on individuals with musculoskeletal complaints [5, 32] and mental health disorders [9]. The key element of both the inpatient and outpatient program in the present study was ACT. Differences between the two programs, in addition to the inpatient versus outpatient setting, were that the inpatient program was more extensive and included physical training, creation of a return to work plan and a workplace visit in 13 % of the cases. However, these additional components did not induce additional benefits.

Most occupational rehabilitation programs described in the scientific literature use some sort of cognitive behavioral therapy approach [6, 11]. In ACT the participants are encouraged to accept pain rather than try to control it. It has therefore been argued that pain might not be the best outcome measure for acceptance-based therapies [33]. This also applies to several of the other outcomes in this study like anxiety and depression, as ACT emphasize behavior change and not symptom reduction [34]. This is line with our findings of modest changes for these outcomes. The outpatient program was slightly more effective in reducing one pain variable, but the difference was not clinically significant and due to the number of statistical tests performed this result should be interpreted with caution.

We found an increase in health related quality of life in both groups measured by 15D, estimated to be 0.03 (95 % 0.01–0.06) for the inpatient program and 0.05 (95 % CI 0.02–0.07) for the outpatient program. The clinical importance of this change is uncertain, but it is in the area of cut-off suggested as a minimum important change [30]. When this is compared to the rather small changes observed on the other measures this might suggest that the focus of ACT on values and acceptance of negative experiences in life might have changed how the participants perceive their quality of life despite little change in health symptoms.

Few randomized studies have included participants with different diagnoses in the same return to work programs. As we included individuals on sick leave due to musculoskeletal, mental or general/unspecific disorders, some had pain and others not, which was also the case for anxiety and depression symptoms. This would likely reduce the statistical power to detect between group effects. However, we performed subgroup analyses according to the participants` main sick-leave diagnosis. Participants with a psychological diagnosis had a somewhat larger reduction in depression symptoms than participants with a musculoskeletal diagnosis. However, there was no difference between participants with musculoskeletal and psychological diagnoses in reductions of average pain. As a substantial degree of overlap in symptoms is common in these patients [35, 36] and the diagnostic labelling by the general practitioner may be somewhat arbitrary [37], we performed subgroup analyses for highest baseline scores on anxiety and depression symptoms and average pain. The estimates were fairly similar to the main analyses. It should be noted that the post hoc subgroup analyses were not planned a priori.

The main strength of this randomized study was that all participants were invited from the Social Security System Registry, meaning there was no referral bias. Return to work rehabilitation centers have existed for about 30 years in Norway, but this is the first randomized controlled study investigating effects on somatic or mental health of such programs. The programs were not diagnosis specific and add important knowledge to a field where previous research has focused on diagnosis specific interventions. Also, the study included a broad range of validated health-related measures.

Some limitations should be addressed. Firstly, the response rate for the questionnaires were low at 3, 6 and 12 months. At the start and the end of the programs, more people answered the questionnaires in the inpatient program than in the outpatient program. The participants in the inpatient program answered the questionnaire at the rehabilitation center, while the outpatient participants did it at home. During follow-up, questionnaires were answered at home for both groups and the numbers of missing questionnaires were similar. We therefore assume that the structural differences in collecting questionnaire data account for the differences in responses between the two groups at the start and end of the intervention. For analyses we used linear mixed models which are less sensitive to missing values in outcome data. Still, these models rely on the assumption of “missing at random”, and we cannot disregard the possibility of bias due to loss to follow-up. However, we consider it unlikely that such bias should influence the two groups differentially and thereby the main results of the study. This assumption is strengthened by register-based sick leave data showing a similar number of sick leave days during 12 months of follow-up between participants responding/not responding to the questionnaire at 12 months.

In the current study there was no usual care control group. Therefore, we cannot distinguish between the effects of rehabilitation and time. It should also be noted that the power calculation for the study was done with regard to the primary outcome (sickness absence) and not the secondary outcomes presented in this article.

With regards to external validity it should be noted that from the over 3000 invitations sent, only 275 individuals accepted the invitation. A possible explanation might be that they had to be prepared to be away from their family for 2 weeks if randomized to the inpatient program. With only about 8 % of the invited accepting the invitation the generalizability of the results is a challenge. However, it should be noted that only a small portion of people on sick leave in Norway are referred to occupational rehabilitation centers. By inviting participants this broadly we were able to reach all individuals on sick-leave with these diagnoses without referral bias induced by the general practitioner.

## Conclusions

There was no substantial difference between the programs on somatic and mental health; hence, this study presents no support that a 4 + 4 days inpatient multicomponent rehabilitation program is superior to a less comprehensive outpatient program. Whether a longer lasting inpatient program will have greater effects on somatic and mental health will be investigated in an upcoming study.

## Electronic supplementary material

Below is the link to the electronic supplementary material.

Supplementary material 1 (DOCX 29 kb)
