# Supplementary material for: Effects of Inpatient Multicomponent Occupational Rehabilitation versus Less Comprehensive Outpatient Rehabilitation on Somatic and Mental Health: Secondary Outcomes of a Randomized Clinical Trial
Source: J Occup Rehabil. 2016 Nov 4;27(3):456–66. doi: 10.1007/s10926-016-9679-5 (PMC5591353; doi:10.1007/s10926-016-9679-5)
Supplement: Supplementary file 1 — Supplementary material 1 (DOCX 29 kb) [file 10926_2016_9679_MOESM1_ESM.docx]

**Online appendix**

Table 1: Comparison of estimated scores between the inpatient and the outpatient program on self-rated health and function (Coop-Wonca). Means and mean differences with 95% confidence intervals (95% CI) were estimated using linear mixed models (unadjusted model).

|  |  | Inpatient  program | | Outpatient program | | Estimated difference between programs ^b^ | | |
| --- | --- | --- | --- | --- | --- | --- | --- | --- |
|  |  | mean | 95% CI | mean | 95% CI | mean | 95% CI | p-value |
| **Self-rated health** (1-4) | Start of program  End of program  3 months  6 months  12 months | 2.1  2.3  2.4  2.4  2.3 | 2.0- 2.3  2.1- 2.4  2.3- 2.6  2.2- 2-6  2.1- 2.6 | 2.0  2.4  2.4  2.4  2.5 | 1.8- 2.2 2.2- 2.6 2.2- 2.6  2.2- 2.6  2.2- 2.7 | -0.3 | -0.6- 0.1 | 0.14 |
| **Coop-Wonca physical fitness**  (1-5) | Start of program  End of program  3 months  12 months | 2.2  2.5  2.2  2.3 | 2.0- 2.5 2.2- 2.7 1.9- 2.5  2.0- 2.5 | 2.3  2.5  2.0  2.0 | 2.0- 2.5 2.2- 2.7 1.7- 2.3  1.7- 2.3 | -0.3 | -0.7-0.1 | 0.21 |
| **Coop-Wonca feelings** (1-5) | Start of program  End of program  3 months  12 months | 2.5  2.4  2.2  2.4 | 2.2- 2.8 2.1- 2.7 1.9- 2.5  2.1- 2.7 | 2.5  2.4  2.2  2.5 | 2.2- 2.8 2.1- 2.8 1.9- 2.6  2.1- 2.8 | 0.1 | -0.3-0.5 | 0.70 |
| **Coop-Wonca daily activity** ^a^  (1-5) | Start of program  End of program  3 months  12 months | 2.6  2.6  2.3  2.1 | 2.4- 2.8 2.3- 2.8 2.0- 2.6  1.8- 2.4 | 2.7  2.5  2.2  2.1 | 2.4- 2.9 2.2- 2.8 1.9- 2.5  1.8- 2.4 | -0.1 | -0.5- 0.3 | 0.63 |
| **Coop-Wonca social activity**  (1-5) | Start of program  End of program  3 months  12 months  4 | 2.4  2.2  2.1  2.4 | 2.2- 2.7 2.0- 2.5 1.8- 2.4  2.0- 2.7 | 2.4  2.2  2.1  1.9 | 2.1- 2.7 1.9- 2.5 1.8- 2.5  1.5- 2.2 | -0.4 | -0.9- 0.1 | 0.08 |

^a^ Estimates presented are from models without random slope due to lack of convergence

^b^ Estimated from start of program to 12 months after the program; inpatient minus outpatient program

Table 2: Comparison of estimated scores between the inpatient and the outpatient program for HADS anxiety and depression according to sick-leave diagnosis and baseline score. Means and mean differences with 95% confidence intervals (95% CI) were estimated using linear mixed models (unadjusted model).

|  |  | Inpatient  program | | Outpatient program | | Estimated difference between programs ^b^ | | |
| --- | --- | --- | --- | --- | --- | --- | --- | --- |
|  |  | mean | 95% CI | mean | 95% CI | mean | 95% CI | p-value |
| **HADS depression**  (0-21)  Psychiatric diagnosis (P) | Screening  Start of program  End of program  3 months  12 months | 8.8  9.2  8.1  7.2  7.1 | 7.4- 10.2  7.8- 10.6 6.7- 9.6 5.7- 8.8  5.2- 9.0 | 7.3  7.3  5.7  5.3  5.2 | 5.8- 8.9 5.8- 8.9 4.1- 7.3 3.6- 6.9  3.2- 7.2 | -0.1 | -2.9- 2.7 | 0.95 |
| **HADS depression**  (0-21)  Musculoskeletal diagnosis (L) | Screening  Start of program  End of program  3 months  12 months | 5.5  5.4  5.1  4.4  5.0 | 4.4- 6.5 4.3-6.5 3.9- 6.2 3.2- 5.6  3.7- 6.4 | 5.1  4.7  4.6  3.7  3.8 | 3.9- 6.3 3.5- 5.9 3.3- 5.9 2.3- 5.1  2.3- 5.3 | -0.5 | -2.3- 1.2 | 0.55 |
| **HADS depression**  (0-21)  Baseline score ≥ 8 | Screening  Start of program  End of program  3 months  12 months | 10.7  10.3  9.2  7.9  8.7 | 9.9-11.6  9.3-11.2  8.0-10.3 6.5- 9.3  6.8- 10.6 | 10.8  8.8  7.5  6.0  7.3 | 9.6-11.9 7.5-10.1 5.8- 9.1 4.2- 7.9  5.1- 9.5 | 0.0 | -2.7- 2.8 | 0.99 |
| **HADS anxiety** (0-21)  Psychiatric diagnosis (P) | Screening  Start of program  End of program  3 months  12 months | 10.1  10.4  9.7  8.9  8.2 | 8.8-11.3 9.0- 11.7 8.2- 11.2  7.3- 10.6  6.2- 10.1 | 9.3  8.0  7.9  6.3  7.2 | 7.9- 10.7 6.4- 9.5 6.2- 9.5 4.6- 8.1  5.2- 9.3 | 1.5 | -1.0- 3.9 | 0.24 |
| **HADS anxiety** (0-21)  Musculoskeletal diagnosis (L) | Screening  Start of program  End of program  3 months  12 months | 6.4  6.0  5.5  4.9  5.8 | 5.2- 7.6 4.8- 7.2 4.3- 6.8 3.6- 6.2  4.4- 7.2 | 6.1  5.5  6.1  4.8  4.6 | 4.8- 7.4 4.2- 6.8 4.6- 7.5 3.3- 6.4  3.1- 6.2 | -0.7 | -2.5 1.1 | 0.45 |
| **HADS anxiety^a^**  (0-21)  Baseline score ≥ 8 | Screening  Start of program  End of program  4 3 months  12 months | 11.5  10.5  10.0  9.2  9.1 | 10.5- 12.4 9.5- 11.5 8.9-11.1 7.9-10.4  7.9- 10.4 | 11.3  9.6  9.4  8.3  8.9 | 10.2- 12.4 8.4-10.8 8.1-10.8 6.9- 9.8  7.4- 10.3 | 0.6 | -1.4-2.6 | 0.55 |

^a^ Estimates presented are from models without random slope due to lack of convergence

^b^ Estimated from start of program to 12 months after the program; inpatient minus outpatient program

Table 3: Comparison of estimated scores between the inpatient and the outpatient program for pain according to sick-leave diagnosis and baseline score. Means and mean differences with 95% confidence intervals (95% CI) were estimated using linear mixed models (unadjusted model).

|  |  | Inpatient  program | | Outpatient program | | Estimated difference between programs ^a^ | | |
| --- | --- | --- | --- | --- | --- | --- | --- | --- |
|  |  | mean | mean | 95% CI | mean | 95% CI | mean | p-value |
| **Average Pain**  (0-10)  Psychiatric diagnosis (P) | Screening  Start of program  End of program  3 months  12 months | 3.4  3.2  2.8  2.6  2.9 | 2.8- 4.1 2.6- 3.9 2.1- 3.5 1.8- 3.3  1.9- 3.8 | 3.7  3.8  2.9  3.1  2.9 | 2.9- 4.4  3.1- 4.6 2.1- 3.6 2.3- 3.9  1.9- 3.8 | -0.6 | -1.8- 0.7 | 0.38 |
| **Average Pain**  (0-10)  Musculoskeletal diagnosis (L) | Screening  Start of program  End of program  3 months  12 months | 5.7  4.9  4.9  4.2  4.9 | 5.2- 6.2 4.4- 5.5 4.4- 5.5 3.6- 4.9  4.1- 5.7 | 5.5  5.4  5.7  4.5  4.6 | 4.9- 6.1 4.8- 6.0 5.0- 6.4 3.7- 5.3  3.7- 5.4 | -0.8 | -1.9- 0.3 | 0.14 |
| **Average Pain**  (0-10)  Baseline score  ≥ 4 | Screening  Start of program  End of program  3 months  12 months | 5.9  4.9  4.8  4.5  4.9 | 5.6-6.3 4.5-5.3 4.3-5.3 3.9- 5.1  4.1- 5.6 | 5.7  5.4  5.0  4.5  4.4 | 5.3-6.1 5.0-5.9 4.4- 5.6 3.8-5.2  3.6- 5.2 | -1.0 | -2.0-0.0 | 0.61 |

^a^ Estimated from start of program to 12 months after the program; inpatient minus outpatient program
